# Coculture with hiPS-derived intestinal cells enhanced human hepatocyte functions in a pneumatic-pressure-driven two-organ microphysiological system

**DOI:** 10.1038/s41598-021-84861-y

**Published:** 2021-03-08

**Authors:** Marie Shinohara, Hiroshi Arakawa, Yuuichi Oda, Nobuaki Shiraki, Shinji Sugiura, Takumi Nishiuchi, Taku Satoh, Keita Iino, Sylvia Leo, Yusuke Kato, Karin Araya, Takumi Kawanishi, Tomoki Nakatsuji, Manami Mitsuta, Kosuke Inamura, Tomomi Goto, Kenta Shinha, Wataru Nihei, Kikuo Komori, Masaki Nishikawa, Shoen Kume, Yukio Kato, Toshiyuki Kanamori, Yasuyuki Sakai, Hiroshi Kimura

**Affiliations:** 1 Institute of Industrial Science, The University of Tokyo, Tokyo, Japan; 2 Faculty of Pharmacy, Institute of Medical, Pharmaceutical and Health Sciences, Kanazawa University, Kanazawa, Japan; 3 Department of Mechanical Engineering, School of Engineering, Tokai University, Kanagawa, Japan; 4 School of Life Science and Technology, Tokyo Institute of Technology, Kanagawa, Japan; 5 Biotechnology Research Institute for Drug Discovery, National Institute of Advanced Industrial Science and Technology (AIST), Tsukuba, Japan; 6 Advanced Science Research Centre, Kanazawa University, Kanazawa, Japan; 7 Department of Chemical System Engineering, Graduate School of Engineering, The University of Tokyo, Tokyo, Japan

**Keywords:** Biological techniques, Biotechnology

## Abstract

Examining intestine–liver interactions is important for achieving the desired physiological drug absorption and metabolism response in in vitro drug tests. Multi-organ microphysiological systems (MPSs) constitute promising tools for evaluating inter-organ interactions in vitro. For coculture on MPSs, normal cells are challenging to use because they require complex maintenance and careful handling. Herein, we demonstrated the potential of coculturing normal cells on MPSs in the evaluation of intestine–liver interactions. To this end, we cocultured human-induced pluripotent stem cell-derived intestinal cells and fresh human hepatocytes which were isolated from PXB mice with medium circulation in a pneumatic-pressure-driven MPS with pipette-friendly liquid-handling options. The cytochrome activity, albumin production, and liver-specific gene expressions in human hepatocytes freshly isolated from a PXB mouse were significantly upregulated via coculture with hiPS-intestinal cells. Our normal cell coculture shows the effects of the interactions between the intestine and liver that may occur in vivo. This study is the first to demonstrate the coculturing of hiPS-intestinal cells and fresh human hepatocytes on an MPS for examining pure inter-organ interactions. Normal-cell coculture using the multi-organ MPS could be pursued to explore unknown physiological mechanisms of inter-organ interactions in vitro and investigate the physiological response of new drugs.

## Introduction

The liver and intestines are important sites for drug absorption, distribution, metabolism, and excretion (ADME), which affect the drug efficacy and toxicity. In vivo human pharmacokinetics prediction is performed using physiologically based pharmacokinetic (PBPK) models based on the physiological characteristics and drug parameters, such as organ volume and blood flow rate, physicochemical properties, membrane permeability, and metabolic stability. The drug parameters are often obtained from in vitro cell culture models, but the prediction accuracy is generally low. This could be due to a discrepancy between the drug parameters estimated with in vitro models and those estimated in vivo in humans, suggesting there is organ–organ crosstalk. It has been reported that the liver and intestines interact through metabolic reactions^1^. The small intestine and liver contain the metabolic enzymes, cytochrome 3A and *p*-glycoprotein, which affect the drug metabolism^2^. These enzymes work in a similar way, which is the major concern in pharmacokinetics^3^. Nevertheless, the inter-organ interaction mechanisms are still unclear. In vitro studies can focus on evaluating specific liver–intestine interactions using liver and intestine cells. The coculture of HepG2 and Caco-2 cells, performed to mimic the metabolic processes occurring in the liver and intestine, showed possible organ-to-organ interactions in terms of the physiological protection of the human body against exogenous stimul^4,5^. This culture method successfully characterised the intestine–liver interactions mimetic relevance in drug metabolism, such as the cytochrome P450 activity, which is one of the main indices of metabolic characterisation assays^4,6,7^. However, conventional in vitro culture methods can only be performed in non-physiological environments, which limits the validity of the results owing to the low function of the cell line and low regulation of the culture medium flow. Therefore, in vitro culture systems that enable a more accurate examination of the physiological reactions are required to evaluate the organ–organ interactions precisely^8,9^.

Microphysiological systems (MPSs) have been proposed to miniaturize the physiological environment in vivo^10^. Multi-organ MPSs achieve the integration of multiple organ models or tissues and have demonstrated the occurrence of complex and physiological inter-organ reactions^11^, unlike single-organ culture systems, such as liver-on-a-chip^12,13^ and gut-on-a-chip^14,15^ systems. For example, inflammatory inter-organ crosstalk was observed using multi-organ MPSs^16^. However, most of the MPSs are closed systems, complicating the access of the culture chamber in extreme conditions, i.e., the maintenance of cells and collection of samples. Recently, a couple of open-type multi-organ MPSs that allow culture chamber access have been developed^16,17^. Satoh et al. developed another open-type two-organ MPS, namely a pneumatic-pressure-driven two-organ MPS, which is similar in appearance to the multi-well culture plate^18^. This system enables pipette-friendly liquid handling, which is advantageous for medium sampling and replenishment^19^. The culture medium is circulated in two culture chambers with the help of the sequential pneumatic pressure. However, previous studies on multi-organ MPSs used cell lines or primary cryopreserved cells^20–22^. The cell lines and types experienced significant loss of functions in comparison with freshly isolated cells.

The cell source is another issue in the exploration of intestine–liver interactions in in vitro culture systems. Cells freshly isolated from human organs are ideal for in vitro studies. However, primary cells have problems with supply stability and lot-to-lot variation. Cells freshly isolated from humanized animals are expected to constitute a stable cell source. Human hepatocytes freshly isolated from a PXB mouse (hereinafter referred to as PXB-cells) are fresh hepatocytes isolated from a chimeric mouse with a humanized liver, and consist of human hepatocytes in excess of 90% (PXB mouse)^23^. The characteristics of these cells can be determined in the same manner as those of the cryopreserved hepatocytes of clinical samples and they maintain their function for at least 21 days^24^. Furthermore, primary intestinal cells are difficult to culture in vitro. Human-induced pluripotent stem (hiPS) cells are expected to be a promising cell source. Ogaki et al*.* developed hiPS-derived intestinal epithelial cells that exhibited more alkaline phosphatase activity and expressed more drug transporters and metabolic enzymes than a carcinoma-derived cell line Caco-2^25^.

In this study, we aim to accurately evaluate intestine–liver interactions in vitro by coculturing normal cells and maintaining their original organ functions. hiPS derived intestinal cells (hiPS-intestinal cells) and PXB-cells were cocultured in a pneumatic-pressure-driven two-organ MPS to evaluate intestine–liver interactions. The MPS with pipette-friendly liquid handling options allows easy maintenance and sampling of delicate cells, such as primary and hiPS-derived cells. We examined the effect of the coculture on their metabolic functions to demonstrate the applicability of the MPS in discovering inter-organ interactions between the liver and intestine. This study is the first to demonstrate the coculturing of hiPS-intestinal cells and fresh human hepatocytes on an MPS for examining pure inter-organ interactions.

## Results and discussion

### hiPS-intestinal cells maintained their functions in the coculture

**Figure 1:**
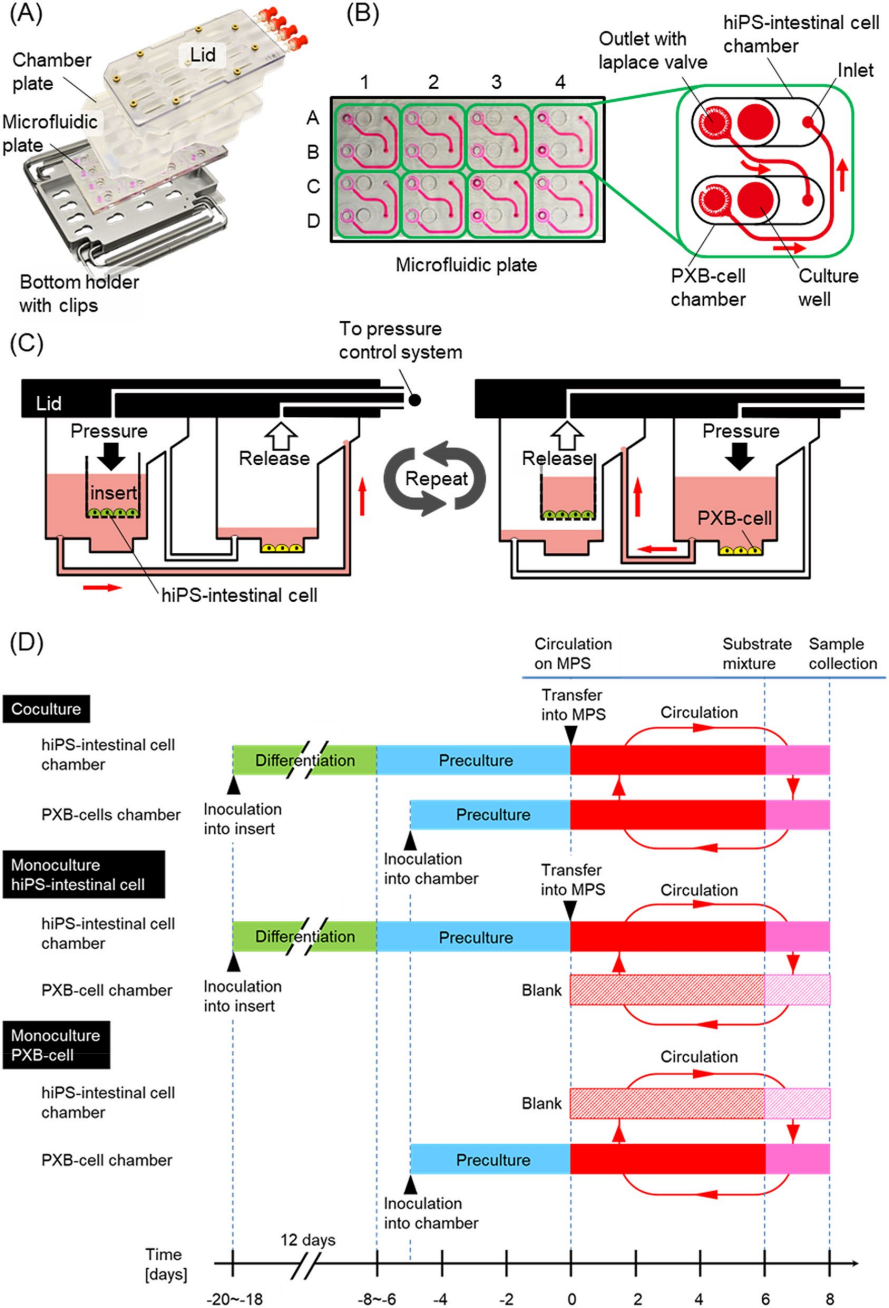
Pressure-driven two-organ MPS used for coculture of hiPS-intestinal cells and PXB-cells. (**A**) Photograph of the two-organ MPS. (**B**) Design of the PDMS microfluidic plate containing eight throughput culture units. Each culture unit consists of the hiPS-intestinal and PXB-cell culture chambers. (**C**) Schematic of the circulation flow direction during cell coculture. (**D**) Experimental schedule for coculture and monoculture.

**Figure 2:**
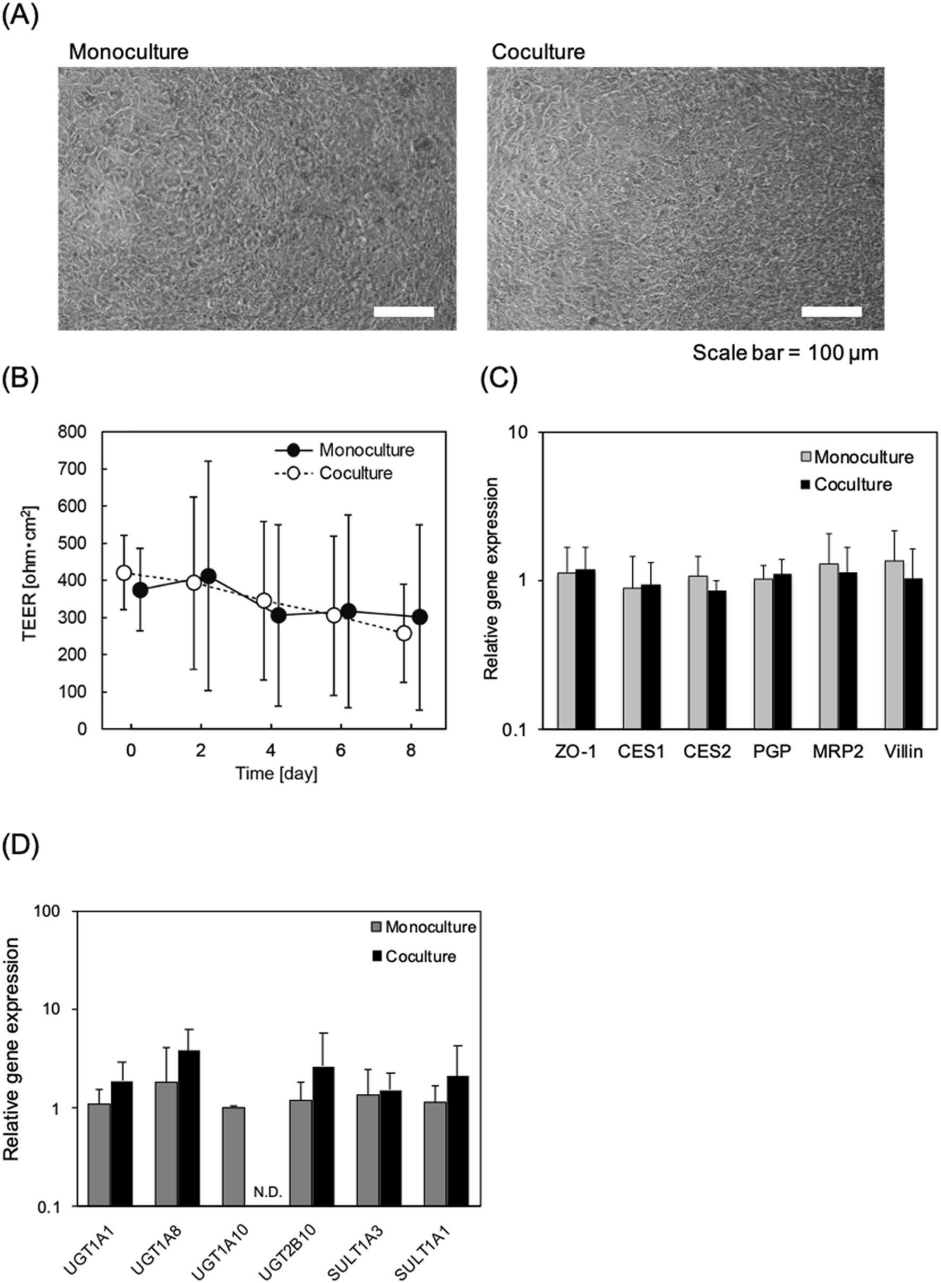
Evaluation of coculture effects in hiPS-intestinal cells. (**A**) hiPS-intestinal cells morphologies in monoculture and coculture. (**B**) Transepithelial electrical resistance (TEER) of hiPS-intestinal cells measured from day 0 to day 8 (Fig. 1D). Each data point represents the mean ± standard deviation (SD, n = 12) from two independent experiments. (**C**) Intestinal gene expression in hiPS-intestinal cells. The relative gene expression in hiPS-intestinal cells was measured using qRT-PCR. Each bar represents the mean ± SD (n = 6–11) from two independent experiments. Each value was normalized to the value obtained from the monoculture for each gene. (**D**) Phase II drug metabolising enzyme related gene expression in hiPS-intestinal cells. The relative gene expression in hiPS-intestinal cells was measured using qRT-PCR. Each bar represents the mean ± SD (n = 3–11) from two independent experiments. Each value was normalized to the value obtained from the monoculture for each gene.

hiPS-intestinal cells and PXB-cells were cocultured for 8 days in the pneumatic-pressure-driven two-organ MPSs shown in Fig. 1 (see “Materials and methods”). From Fig. 2A, it can be seen that the morphology of the hiPS-intestinal cells in monoculture and coculture conditions are very similar. Figure 2B reveals that the transepithelial electrical resistance (TEER) of the hiPS-intestinal cells was also similar in the monoculture and coculture conditions.

The TEER value in primary human intestinal epithelial cell cultures has been reported to be less than 100 Ω cm^2^ and to take more than 2 weeks to stabilize^26^. In this study, the hiPS-intestinal cells sustained high TEER values, indicated that tight junctions of the hiPS-intestinal cells were well maintained. The gene expression of hiPS-intestinal cells related to tight junction formation (ZO-1), metabolism (CES1 and CES2), drug transport (*P*-glycoprotein (PGP) and MRP2) and villus markers (Villin) were examined and no significant difference was observed between the monocultures and cocultures (Fig. 2C). Also, the gene expressions of phase II drug metabolizing enzymes were not significantly different except for UGT1A10 showing decreased expression in coculture (Fig. 2D).

The intestinal cell function marker CYP3A was not detected in the hiPS-intestinal cells in either the coculture or the monoculture conditions; however, an indication of their slight presence was observed after differentiation (data not shown). In this experiment, the culture medium for PXB-cells was used in both the coculture and monoculture conditions. Fujii et al. discovered that human intestinal tissues require insulin growth factor 1 (IGF-1) and fibroblast growth factor 2 (FGF2) for long term culture^27^. Generally, hiPS-derived cells should be maintained carefully in well-optimized culture media for long-term culture after differentiation and coculture to sustain their functions. The hiPS-intestinal cells cultured in the PXB-cell medium may lose some of their original functions owing to culture medium issues. To partly address to these issues, we compared the performances of hiPS-intestinal cells and PXB-cells in three culture mediums; PXB-cell medium (used in the coculture using the MPS), hiPS-intestinal cells medium^28^ and their 1:1 mixed medium. The mRNA expressions of CYPs and drug transporters in hiPS-intestinal cells were higher in hiPS-intestinal cells medium than in the PXB-cells medium or mixed medium except for PGP and CYP1A2 (Fig. S2A). Function of CYP enzymes on hiPS-intestinal cells were also evaluated through the cocktail approach using a liquid chromatography/tandem mass spectrometry (LC–MS/MS) system. Almost similarly to the gene expressions (Fig. S2A), CYP activities were higher by hiPS-intestinal cell medium (Fig. S2B). However, the gene expression of albumin, a representative marker of mature hepatocytes, was undesirably enhanced in hiPS-intestinal cells with hiPS-intestinal cell medium (Fig. S2B). There should be threshold of medium components to maintain intestinal function efficiently while preventing dedifferentiation. Moreover, the mRNA expression of CYP1A1 and CYP1A2 of PXB-cells were higher in hiPS-intestinal cell medium or mixed medium than PXB-cell medium, while that of CYP2A6 and albumin were decreased (Fig. S3). These results suggest that that there should be threshold concentrations of some components in the hiPS-intestinal cell medium to maintain higher intestinal functions while preventing dedifferentiation of once differentiated intestinal cells from iPS cells. Therefore, culture media for coculturing both hiPS-intestinal cells and PXB-cells should be optimized in future studies.

### Functions of the PXB-cells were enhanced in the coculture

**Figure 3:**
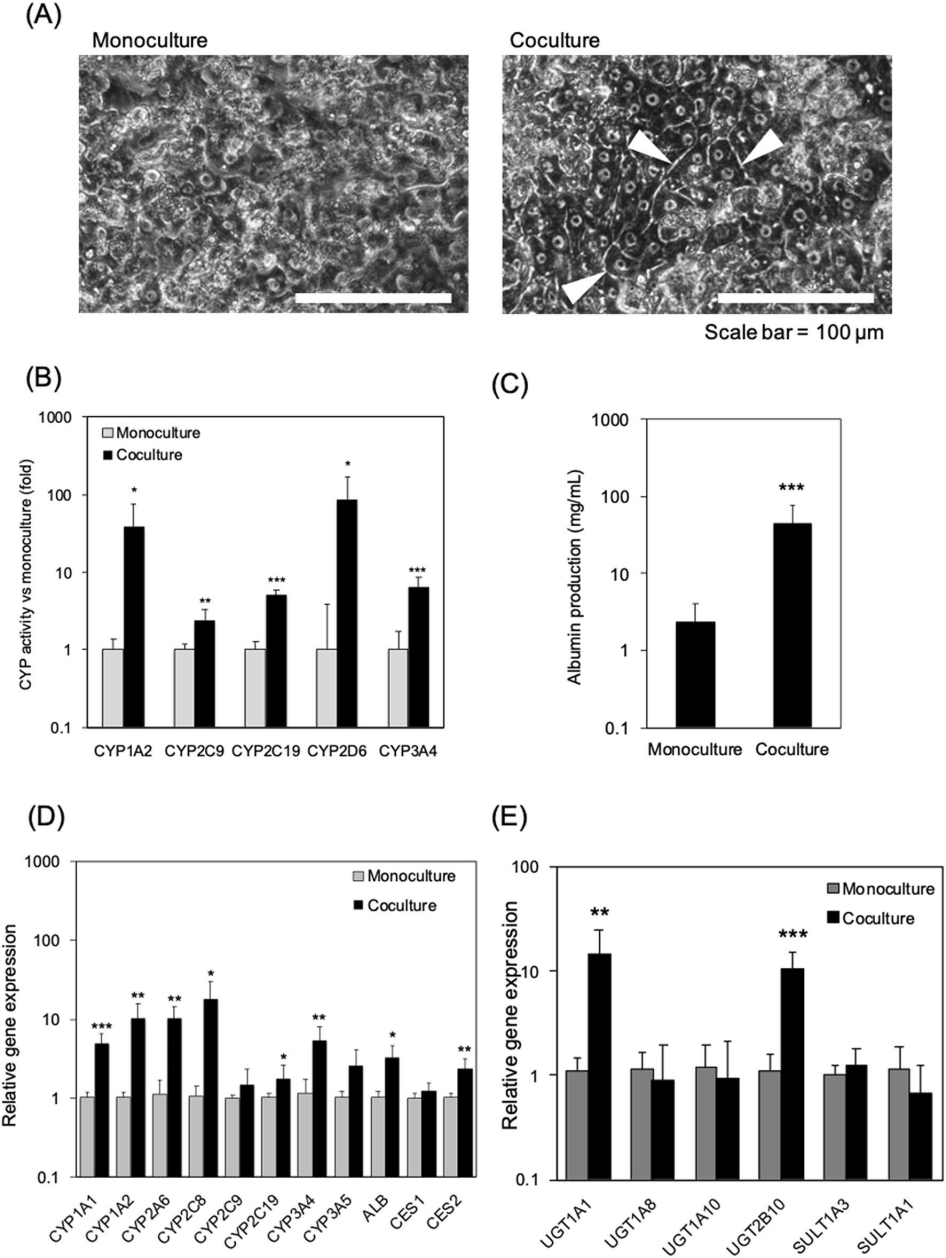
Evaluation on coculture effects in PXB-cells. (**A**) Morphology of PXB cells in monoculture and coculture. The white arrows point to bile canaliculi structures. (**B**) Enhancement in liver function in PXB-cells by coculture with iPS-intestinal cells. The CYP activities using each specific substrate in monoculture and coculture of PXB-cells were measured for 48 h (from day 6 to day 8 in Fig. 1D). Each bar represents the mean ± SD (n = 8) from two independent experiments normalized to the value obtained from the monoculture. (**C**) Albumin concentration in culture medium measured on day 8 (Fig. 1D). Each bar represents the mean ± SD (n = 11) from two independent experiments. (**D**) Relative gene expression of PXB-cells. Each data point represents the mean ± SD (n = 7) from two independent experiments. Each value is normalized to the value obtained from the monoculture of each tested gene. In all panels, *p < 0.05, **p < 0.01, and ***p < 0.001. (**E**) Phase II drug metabolising enzyme related gene expression in PXB-cells. Each data point represents the mean ± SD (n = 10–11) from two independent experiments. Each value is normalized to the value obtained from the monoculture of each tested gene. In all panels, *p < 0.05, **p < 0.01, and ***p < 0.001.

Figure 3A shows the morphology of the PXB-cells in the monoculture and coculture conditions. It can be seen that a bile canaliculi structure is formed between the cell–cell boundary of the hepatocytes. Small morphological differences are observed in the two conditions; most PXB-cells exhibit a clear cell–cell boundary and well-formed bile canaliculi structures after 8 days of coculture on the MPS, whereas the boundary and bile canaliculi structures are observed in the monoculture along with cellular debris and swelling. This suggests that the PXB-cells were better maintained in the coculture conditions with hiPS-intestinal cells than in the monoculture conditions.

To reveal the effect of the coculture of hepatic and intestinal cells on metabolic enzymes, the metabolic activities by CYP enzymes on PXB-cells were evaluated through the cocktail approach using a liquid chromatography/tandem mass spectrometry (LC–MS/MS) system. The metabolic activities of CYP1A2, CYP2C9, CYP2C19, CYP2D6, and CYP3A4 in coculture were enhanced when compared with those in monoculture (Fig. 3B), while those of CYP2A6, CYP2B6, and CYP2C8 were not detected in all culture conditions. Because no metabolic activities were observed with any CYP enzymes in the hiPS-intestinal cells (data not shown), the metabolic activities observed in the cocultures were assumed to be the enhancement of those in the PXB-cells in this system. Moreover, the concentration of albumin in the coculture medium was 22 higher than that in the monoculture medium (Fig. 3C). These results suggested that the hepatic function was improved by coculture with intestinal cells.

To elucidate the mechanism of the enhancement in the CYP activities, we measured the mRNA expression of CYP enzymes in the PXB-cells in coculture and monoculture conditions. The mRNA expression of CYP1A2, CYP2A6, CYP2C8, CYP2C19, CYP3A4, ALB, and CES2 was found to be significantly higher in the coculture condition, while that of CYP2C9 and CYP3A5 was only slightly higher (Fig. 3D). We also measured the mRNA expression of phase II drug metabolizing enzymes (Fig. 3E). While mRNA expressions of UGT1A8, UGT1A10, SULT1A3, and SULT1A1 were not significantly different, those of UGT1A1 and UGT2B10 were enhanced in the coculture condition. We argue that the higher metabolic activity of the PXB-cells in coculture is due to transcription because the xenobiotic metabolism and transcriptional variation of CYP enzymes are regulated by the constitutive androstane receptor (CAR) and pregnane X receptor (PXR)^29^.

On the other hand, the adsorption of several drugs (coumarin, bupropion, amodiaquine, bufuralol, and midazolam) was observed in the MPS without cells, while adsorption of phenacetin, dicrophenac and mephenytoin was not observed (Fig. S1). One reason why the metabolic activity of several enzymes, such as CYP2A6, CYP2B6, and CYP2C8, was not detected might be the adsorption of substrates on the microfluidic plate composed of polydimethylsiloxane (PDMS), where nonspecific binding is often problematic^30^. The metabolic activity of drugs that are metabolised by hepatocytes faster than the adsorption in PDMS during medium circulation should be measured using LC–MS^31^. PDMS is advantageous because it is easy to fabricate and allows gas permeation, which enhances cell proliferation and function^32^. Therefore, for future studies, it is necessary to design the MPS with materials that overcome issues due to the PDMS.

### Significant protein production may indicate intestine–liver interactions

**Figure 4:**
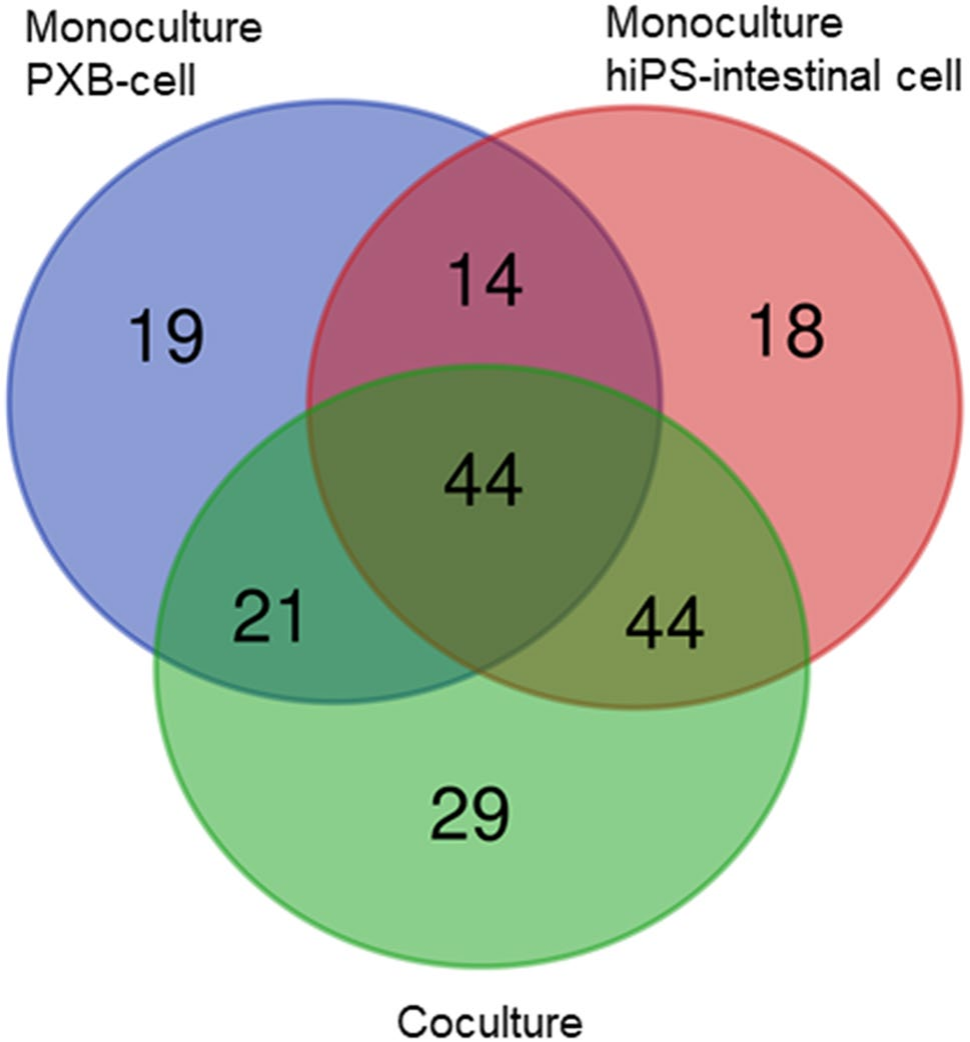
Venn diagram of proteins identified from hiPS-intestinal cell monoculture, PXB-cell monoculture, and coculture samples with LC–MS/MS.

Several kinds of nuclear receptors and transcriptional factors have been reported to be involved in the mRNA expression of CYPs in the liver. For example, mRNA expression of CYP3A4 was found to be regulated by pregnane X receptor, CAR, vitamin D receptor (VDR), and hepatocyte nuclear factor 4α (HNF4α)^30^. The upregulation of these factors might lead to an increase in the enzymatic activities of PXB-cells by coculture with hiPS-intestinal cells. We performed a preliminary proteomic analysis using circulated medium in monoculture and coculture conditions to evaluate the interaction between intestinal-cells and PXB-cells and the differences in the protein synthesis (Fig. 4; Table S1). The raw data has been deposited in the ProteomeXchange Consortium via the Japan ProteOme Standard Repository/Database (ID: PXD017440). The analysis revealed that the expression of 120, 98, and 138 kinds of protein was increased, when compared with non-treatment medium, by more than two times in the hiPS-intestinal cell monoculture, PXB-cell monoculture, and coculture conditions, respectively. For example, the protein expression of apolipoproteins was increased in the medium of the hiPS-intestinal cell monoculture and coculture, suggesting that the hiPS-intestinal cells secreted lipoproteins, such as high density lipoprotein (HDL) and chylomicron, reflecting the in vivo intestinal function^30^. The proteins secreted from hiPS-intestinal cells might increase the mRNA expression of CYPs in PXB-cells. Although the mechanism of the increase in the mRNA expression of the CYP enzymes is not clarified in this study, the results clearly indicate that there is an inter-organ communication between the intestine and liver through an unknown transcriptional mechanism. Further studies with western blotting and functional analyses are required to confirm these proteomic results and the interaction between intestinal cells and PXB-cells.

The coculture of PXB-cells and hiPS-intestinal cells using a two-organ MPS had a positive effect on the liver functions, suggesting that the intestine and liver interact through the physiological metabolism. The culture in the two-organ MPSs enables the comprehensive evaluation of the assays in a dynamic environment. The detailed mechanism of enhanced liver functions could be determined by metabolomics and transcriptomic analyses in a chronological manner. Although further studies on the function of hiPS-intestinal cells is also required, this study demonstrated the availability of organ-to-organ interactions, especially liver–intestine interactions using normal cells instead of cell lines a simple coculture system in MPSs.

## Conclusions

We performed cocultures of hiPS-intestinal cells and PXB-cells with a pneumatic-pressure-driven MPS for two-organ cultures. Although the gene expression and cellular tight junctions were not altered in the hiPS-intestinal cell cultures, the hiPS-intestinal cells maintained their intestinal markers (PGP, MPR2, and Villin). The metabolic gene expressions of the PXB-cells and the albumin production were higher in the cocultures than in the monocultures. The cytochrome activities of the PXB-cells examined with the substrates were also enhanced in the cocultures with hiPS-intestinal cells. This study demonstrated the potential application of intestine–liver organ derived tissue perfusion cultures by using two-organ MPSs for the investigation of intestine–liver interactions in cell-based assays. Further studies using the intestinal tissue model considering enterohepatic circulation are expected to achieve a physiological intestine–liver organ culture system.

## Materials and methods

### Pneumatic-pressure-driven two-organ MPS

The pneumatic-pressure-driven two-organ MPS was used for the coculture of intestinal cells and hepatocytes with medium circulation. The two-organ MPS^18^ was assembled with an aluminium bottom holder with stainless steel clips, a PDMS microfluidic plate, a polycarbonate chamber plate, and glass and polycarbonate lid parts, as depicted in Fig. 1A. There are eight throughput culture units. Each culture unit consists of two culture chambers for culturing hiPS-intestinal cells and PXB-cells (Fig. 1B). The PXB-cell culture chamber has a culture well (6 mm diameter) treated with poly-l-lysine for adhesive cell cultures. A culture insert can also be inserted after removing its hanging parts^18^. We used ad-MED vitrigel (24 well, KANTO CHEMICAL CO., INC., Japan) as the culture insert after cutting two of three hanging parts. The PDMS microfluidic plate, which was fabricated by injection moulding (MEIHO, Japan), contained microfluidic flow channels (298 μm depth and 1000 μm width) and narrow Laplace valves (27 μm depth and 110 μm width). The culture medium was circulated in a microfluidic flow channel in the microfluidic plate; it flowed into the chamber from the elevated inlet and flowed out through the outlet. The semi-one-way medium circulation was generated by a sequentially applied pneumatic-pressure based on the elevated inlet and the narrow outlet Laplace valve using a pressure control system (ASTF0401, Engineering System Co., Ltd., Japan) (Fig. 1D)^18^. The volumes of the inlet to outlet, culture well, culture insert, and culture chamber flow channel were 50, 50, 200, and 250 μL, respectively. A single culture unit consists of two single-organ chambers and requires 900 μL of culture medium in total for the two flow channels, two culture wells, one culture insert, and two culture chambers.

### Differentiation of intestinal cells from human iPS cells

Human iPS cells (hiPS-intestinal cells) and intestinal cells were differentiated by following the methodology outlined in previous studies^25,33^. Human iPS cell line RpChiPS771 (REPROCELL Inc., Japan) was used. Undifferentiated iPS cells were maintained in AK02 StemFit media (Ajinomoto Co. Inc., Japan) on cell culture dishes (Thermo Fisher Scientific K. K., USA) pre-coated with Synthemax II (Corning Inc., USA). Then, RpChiPS771 cells were plated onto M15 cell-precultured dishes and cultured in a methionine deprivation medium (StemFit KA01 media, Ajinomoto Co. Inc., Japan) for five hours to prepare the cells for differentiation by changing their metabolism^33,34^. The medium was transformed to an endoderm differentiation medium M1, supplemented with 3 μM CHIR99021 (FUJIFILM Wako Pure Chemical Corporation, Japan) for 1 day, and changed to M1 without CHIR99021 and cultured for two additional days. M1 consists of DMEM, 4.5 g/L of glucose, 1% non-essential amino acids (NEAA, Thermo Fisher Scientific K. K., USA), 1% l-glutamine (L-Gln, NACALAl TESQUE,INC., Japan), 1% Penicillin, Streptomycin solution (PS; NACALAl TESQUE,INC., Japan), 0.1 mM mercaptoethanol (2-ME, Sigma-Aldrich Co. LLC., USA), serum-free B27 supplement (Thermo Fisher Scientific K. K., USA), and 100 ng/mL recombinant human activin A (Cell Guidance Systems Ltd, UK). After 3 days of culture, endodermal cells were collected, frozen at 2.0 × 10^6^ cells/mL in Bambanker hRM (Nippon Genetics Co., Ltd., Japan), and stocked in liquid N_2_ until use^26^.

For intestinal differentiation, frozen endodermal cells were freeze-thawed and plated onto a rehydrated vitrigel membrane with 24 well inserts (ad-MED VitrigelTM 2, KANTO CHEMICAL CO., INC., Japan, culture area: 0.33 cm^2^/insert) at a concentration of 8.0 × 10^4^ cells/well with M2^28^. The volume of the media was 200 μL for the upper layer and 500 μL for the lower layer of the transwell. M2 consists of DMEM (Thermo Fisher Scientific K. K., USA) supplemented with NEAA, L-Gln, PS, 0.1 mM 2-ME, 1 mg/mL d-Glucose, 10% KnockOut Serum Replacement (Thermo Fisher Scientific K. K., USA), 5 µM 6-Bromoindirubin-3′-oxime (Bio, FUJIFILM Wako Pure Chemical Corporation, Japan), and 10 µM *N*-[*N*-(3,5-difluorophenacetyl-l-alanyl)]-*S*-phenylglycinetert.butyl ester (DAPT, FUJIFILM Wako Pure Chemical Corporation, Japan). Over a 12-day period (day −20 to − 18 to − 8 to − 6 in Fig. 1D), the media in both upper and lower layers was replaced every 2 days with fresh medium and growth factors^25^. Prior to coculture with hepatocytes, hiPS-intestinal cells were cultured in Cellartis Hepatocyte Maintenance Medium (Takara Bio Inc., Japan) for 6–8 days (day − 8 to − 6 to day 0 in Fig. 1D) as preculture. Then, the inserts were set on a pneumatic-pressure-driven MPS (on day 0 in Fig. 1D). The inserts were kept culturing in a normal multiwell plate when the effect of culture medium was evaluated.

### PXB-cell culture

PXB-cells were obtained from PhoenixBio Co. Ltd. in a T-flask. The cells were cultured in DMEM (Thermo Fisher Scientific K. K., USA) containing 10% FBS (Japan Bioserum Co. Ltd., Japan), 20 mM 1-piperazineethanesulfonic acid (HEPES) (Sigma-Aldrich Co. LLC., USA), 44 mM NaHCO_3_ (Sigma-Aldrich Co. LLC., USA), 1% antibiotic–antimycotic (Anti–Anti) solution (Thermo Fisher Scientific K. K., USA), 15 μg/mL L-proline (Sigma-Aldrich Co. LLC., USA), 50 nM dexamethasone (Sigma-Aldrich Co. LLC., USA), 5 μg/mL mouse epidermal growth factor (EGF) (FUJIFILM Wako Pure Chemical Corporation, Japan), 0.1 mM l-ascorbic acid 2-phosphate (Sigma-Aldrich Co. LLC., USA), and 0.25 μg/mL human insulin (Sigma-Aldrich Co. LLC., USA). PXB-cells were dissociated from a T-flask with 0.25% trypsin–EDTA and seeded at a density of 2 × 10^5^ cells/cm^2^ on a culture well that was precoated with collagen type I (Nitta Geratin Inc., Japan) in a pneumatic-pressure-driven MPS (Fig. 1C). The medium was changed every 2 days for 5 days (day − 5 to day 0 in Fig. 1D). PXB cells were kept culturing in a normal multiwell plate when the effect of culture medium was evaluated.

### Coculture of hiPS-intestinal cells and PXB-cells

A two-organ MPS with precultured hiPS-intestinal and PXB-cells was connected to the pneumatic-pressure control system on day 0 (Fig. 1D). The medium was circulated through a 2.2–3.0 kPa sequential pressure (300 s steps), as in a previous study^18^. The flow rate between each culture chamber was estimated to be about 100 μL/min in the experiment. The monoculture condition, in which only hiPS-intestinal cells or PXB-cells were cultured under the medium circulation, was tested as the control experiment (“monoculture hiPS-intestinal cell” and “monoculture PXB-cell” in Fig. 1D). The culture medium for the PXB-cells was used for the coculture and monoculture conditions in the two-organ MPS for eight days (day 0–day 8 in Fig. 1D). The culture medium between the connected two-organ MPS (250 μL for the culture chamber and 200 μL for the insert) was changed every two days. The TEER of the hiPS-intestinal cells was measured every two days.

### Quantitative reverse transcription polymerase chain reaction (RT-qPCR) analyses

RNA from the hiPS-intestinal and PXB-cells in monoculture and coculture was isolated on day 8 and purified with a ReliaPrep RNA cell Miniprep System (Promega corporation, USA) according to the manufacturer instructions. Purified RNA samples were analysed qualitatively and quantitatively with the NanoDrop spectrophotometer (SHIMADZU CORPORATION, Japan). Purified RNA (100 ng) from each sample was reverse transcribed by PrimeScrip Reverse Transcriptase (Takara Bio Inc., Japan). The transcribed complementary DNA samples were evaluated for their gene expressions using SYBR Green (TOYOBO CO., LTD., Osaka, Japan) and detected with the StepOne Plus RT-PCR system (Thermo Fisher Scientific K. K., USA) using each specific primer (Table S2).

### CYP activity evaluation based on the quantitation of metabolites with high-performance liquid chromatography/tandem mass spectrometry (LC–MS/MS)

hiPS-intestinal and PXB-cells were cultured with a substrate mixture containing 20 μM phenacetin, 1 μM diclofenac, 2 μM coumarin, 5 μM bupropion, 0.1 μM amodiaquine, 40 μM mephenytoin, 5 μM bufuralol, 2 μM midazolam, and 1 μM 7-OH coumarin for CYP1A2, CYP2A6, CYP2B6, CYP2C8, CYP2C9, CYP2C19, CYP2D6, CYP3A4, and UGT1A1 respectively^35^ from day 6 to day 8 (Fig. 1D). After culture with the substrate mixture for 48 h, culture medium aliquots (30 µL) were mixed with 5 µL of water and 115 µL of methanol containing 1 µM of niflumic acid as an internal standard. After vortex mixing, the solutions were centrifuged at 21,500×*g* for 5 min at 4 °C, and the resulting supernatants were injected into the LC–MS/MS system. The amount of each metabolite was determined using an LCMS8050 triple quadrupole mass spectrometer (SHIMADZU CORPORATION, Japan) coupled with an LC-30A system (SHIMADZU CORPORATION, Japan). Chromatography was performed on a CAPCELL PAK C18 MG III column (ID 2.0 × 50 mm; Osaka Soda Co. Ltd., Osaka, Japan) at 50 °C through step-gradient elution with a flow rate of 0.4 mL/min according to the following program: 0–0.5 min, 95% A/5% B; 0.5–3.0 min, 95% A/5% B to 20% A/80% B; 3.0–4.0 min, 20% A/80% B; 4.0–4.1 min, 20% A/80% B to 95% A/5% B; 4.1–5.5 min, 95% A/5% B; (A, water containing 0.1% formic acid; B, acetonitrile containing 0.1% formic acid). The detected mass numbers and collision energy (CE) were as follows; acetaminophen for CYP1A2 (152.0 > 110.0, CE: − 9 V), 7-hydroxycoumarin for CYP2A6 (163.0 > 107.0, CE: − 24 V), hydroxybupropion for CYP2B6 (256.0 > 238.0, CE: − 13 V), *N*-desethylamodiaquine for CYP2C8 (328.0 > 283.0, CE: − 18 V), 4′-hydroxydiclofenac for CYP2C9 (312.0 > 230.0, CE: − 32 V), 4′-hydroxymephenytoin for CYP2C19 (235.1 > 150.1, CE: − 17 V), 1′-hydroxybufuralol for CYP2D6 (278.0 > 186.0 CE: − 19 V), 1′-hydroxymidazolam for CYP3A4 (342.0 > 203.0, CE − 27 V), 7-hydroxycoumarin glucuronide for UGTs (339.4 > 163.1, CE: − 18 V), and niflumic acid (283.2 > 265.2, CE: − 21 V). The analytical standard curve for each metabolite was made using commercially available products. Lab solutions software (version 5.89, SHIMADZU CORPORATION, Japan) was used for data manipulation. The productive metabolites of the monoculture of PXB-cells were used to standardize those in the coculture.

### Albumin production measurement

The amount of secreted albumin in the culture medium was measured by a sandwich-type, enzyme-linked immunosorbent assay. A goat anti-human albumin (Bethy Laboratories, Inc., USA) was used as the primary antibody, and a horseradish peroxidase-conjugated goat anti-human albumin (Bethy Laboratories, Inc., USA) was used as the secondary antibody.

### Proteome analysis using nano-liquid chromatography mass spectrometry 

The methods are described in detail in Supplemental information.

### Statistical analysis

The results were expressed as the mean ± standard deviation of the mean (SD). The Student’s two-tailed t test was performed to assess the statistical significance of the results. A *p* value below 0.05 was considered statistically significant.

## Supplementary Information

Supplementary Information 1.

Supplementary Information 2.
