# Supplementary material for: Coculture with hiPS-derived intestinal cells enhanced human hepatocyte functions in a pneumatic-pressure-driven two-organ microphysiological system
Source: Sci Rep. 2021 Mar 8;11:5437. doi: 10.1038/s41598-021-84861-y (PMC7940409; doi:10.1038/s41598-021-84861-y)
Supplement: Supplementary file 1 — Supplementary Information 1. [file 41598_2021_84861_MOESM1_ESM.docx]

Coculture with hiPS-derived intestinal cells enhanced

human hepatocyte functions in a pneumatic-pressure-driven two-organ microphysiological system

Marie Shinohara^1^, Hiroshi Arakawa^2^, Yuuichi Oda^1,3^, Nobuaki Shiraki^4^, Shinji Sugiura^5^, Takumi Nishiuchi^6^, Taku Satoh^5^, Keita Iino^4^, Sylvia Leo^4^, Yusuke Kato^4^, Karin Araya^2^, Takumi Kawanishi^2^, Took Nakatsuji^2^, Manami Mitsuta^7^, Kosuke Inamura^7^, Tomomi Goto^3^, Kenta Shinha^3^, Wataru Nihei^3^, Kikuo Komori^7^, Masaki Nishikawa^7^, Shoen Kume^4^, Yukio Kato^2^, Toshiyuki Kanamori^5^, Yasuyuki Sakai^7^, Hiroshi Kimura^1,3^*

^1^ Institute of Industrial Science, The University of Tokyo, Tokyo, Japan

^2^ Faculty of Pharmacy, Institute of Medical, Pharmaceutical and Health Sciences, Kanazawa University, Kanazawa, Japan

^3^ Department of Mechanical Engineering, School of Engineering, Tokai University, Kanagawa, Japan

^4^ School of Life Science and Technology, Tokyo Institute of Technology, Kanagawa, Japan

^5^ Biotechnology Research Institute for Drug Discovery, National Institute of Advanced Industrial Science and Technology (AIST), Tsukuba, Japan

^6^ Advanced Science Research Centre, Kanazawa University, Kanazawa, Japan

^7^ Department of Chemical System Engineering, Graduate School of Engineering, The University of Tokyo, Tokyo, Japan

*Corresponding author

E-mail: hkimura@tokai-u.jp (HK)

**Supplementary information**

**Materials and Methods**

**Proteome analysis using nano-liquid chromatography mass spectrometry**

Culture medium in monoculture and coculture was collected from the culture chamber on day 8. The culture medium was mixed with four times larger volume of ice-cold acetone. After incubating on ice for one hour, samples were centrifuged at 15,000 ×g at 4 ^o^C. The pellets were resuspended in the mixed buffer of 6 M Urea and 50 mM triethylammonium bicarbonate (TEAB) in pH 8.5. Proteins (50 µg) were adjusted to a final volume of 10 µL, after reduction with 5 mM tris(2-carboxyethyl)phosphine (TCEP) for 30 min at 37 °C in a dark place following alkylation by 24 mM iodoacetamide for 30 min at room temperature in a dark place. Alkylated proteins were digested with 100 ng/ µL trypsin (Promega, Japan) in the ratio of 10:1 at 37 °C for 16 h. Peptides were desalted with Stage tip #84850 (Thermo Pierce, Japan) and acidified with 1% trifluoroacetic acid.

The trypsin-digested peptides were loaded onto the LC system (EASY-nLC 1200; Thermo Fisher Scientific, USA) equipped with a trap column (Acclaim PepMap 100 C18 LC column, 3 µm, 75 µm × 20 mm; Thermo Fisher Scientific), equilibrated with 0.1% formic acid, and eluted with a linear acetonitrile gradient (0-35%) in 0.1% formic acid at a flow rate of 300 nL min^-1^. The eluted peptides were loaded and separated on the column (PepMap C18 LC column, 3 µm, 75 µm x 150 mm; Thermo Fisher Scientific) with a spray voltage of 2 kV (Ion Transfer Tube temperature: 275 ^o^C).　The peptide ions were detected using MS (Orbitrap QE plus MS; Thermo Fisher Scientific) in the data-dependent acquisition mode with the installed Xcalibur software (version 4.0; Thermo Fisher Scientific). Full-scan mass spectra were acquired in the MS over 375-1,500 m/z with a 70,000 resolution. The most intense precursor ions were selected for collision-induced fragmentation in the linear ion trap.

The MS/MS searches were carried out using SEQUEST HT search algorithms against the Homo sapiens (Swiss prot. Tax ID 9609) protein database (2017-9-14) using Proteome Discoverer (PD) 2.2 (Version 2.2.0.388; Thermo Fisher Scientific). Label-free quantification was also performed with PD 2.2 using precursor ions quantifier nodes. The processing workflow included spectrum files RC, spectrum selector, SEQUEST HT search nodes, percolator, ptmRS, and minor feature detector nodes. Oxidation of methionine was set as a variable modification and carbamidomethylation of cysteine was set as a fixed modification. Mass tolerances in MS and MS/MS were set at 10 ppm and 0.6 Da, respectively. Trypsin was specified as protease and a maximum of two missed cleavages were allowed. Target-decoy database searches used for false discovery rate (FDR) calculation and peptide FDR identification were set at 1%.

Label-free quantification was also performed with PD 2.2 using precursor ions quantifier nodes. The consensus workflow included MSF files, Feature Mapper, precursor ion quantifier, PSM groper, peptide validator, peptide and protein filter, protein scorer, protein marker, protein FDR validator, protein grouping, and peptide in protein. Normalization of the abundances was performed using total peptide amount mode.


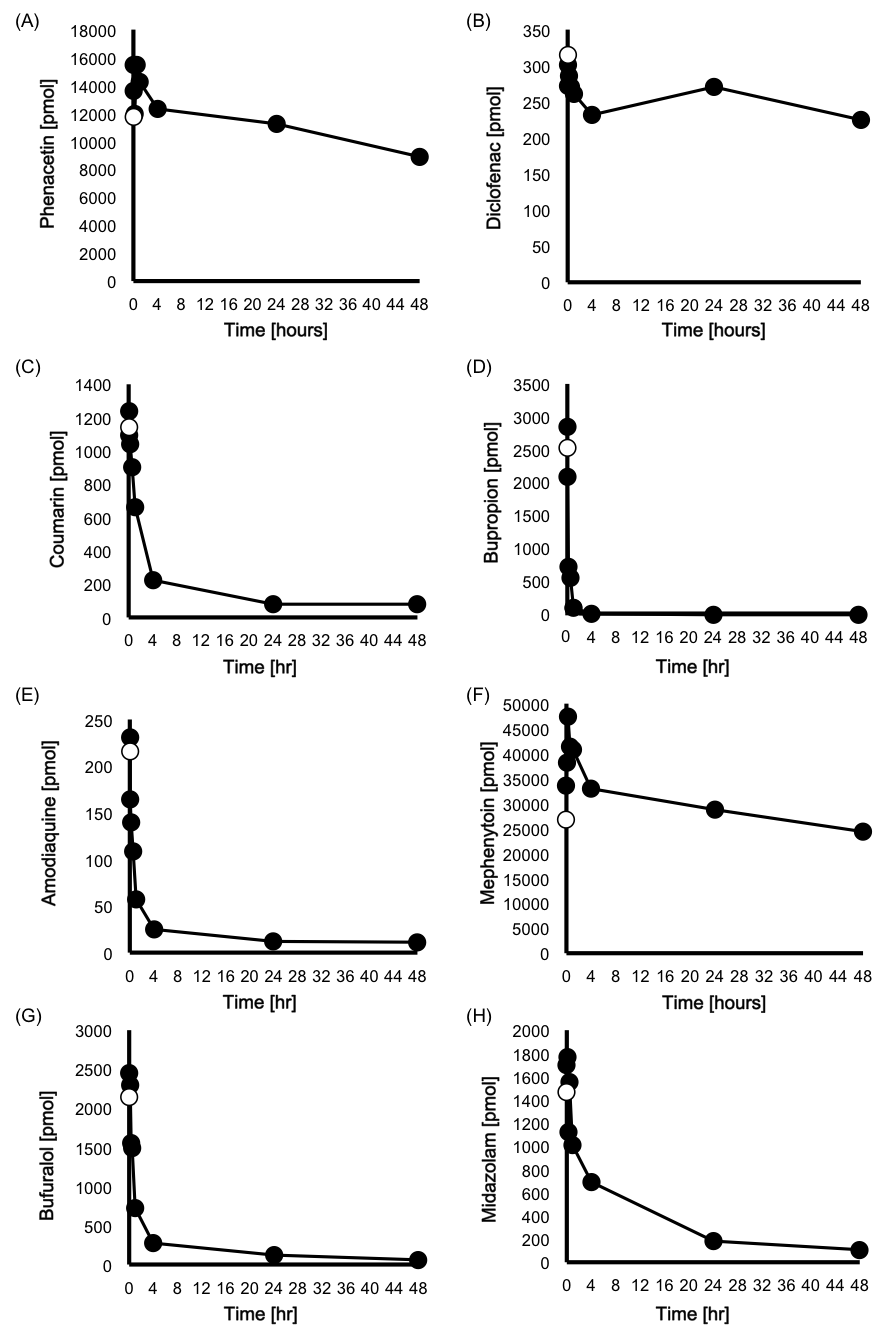


**Supplementary Fig. S1. Adsorption of drugs in PDMS.** Absorption of (A) phenacetin, (B) diclofenac, (C) coumarin, (D) bupropion, (E) amodiaquine, (F) mephenytoin, (G) bufuralol, and (H) midazolam. The white dots represent the concentration of the substrate added to the MPS.

**Supplementary Fig. S2. Evaluation of culture medium effects in hiPS-intestinal cells.**

**Supplementary Fig. S3. Evaluation of culture medium effects on gene expressions in PXB-cells.**

**Supplementary Table S2. Primer sequences.**

|  | forward | reverse |
| --- | --- | --- |
| β-actin | 5’-CCTCATGAAGATCCTCACCGA-3’ | 5’-TTGCCAATGGTGATGACCTGG-3’ |
| ZO-1 | 5’-TGAGGCAGCTCACATAATGC-3’ | 5’-GGTCTCTGCTGGCTTGTTTC-3’ |
| CES1 | 5’-CACTCCTGCTGACTTGACCA-3’ | 5’-CATCCCCTGTGCTGAAGAAT-3’ |
| CES2 | 5’-CATGTTTGTGATCCCTGCAC-3’ | 5’-TGCTTAGCTGCTCCTCTTCC-3’ |
| PGP | 5’-CTTGGCTCTGCTGACCTTTC-3’ | 5’-CAGGACGCCTCTTATTGCTC-3’ |
| MRP2 | 5’-ACAGAGGCTGGTGGCAACC-3’ | 5’-ACCATTACCTTGTCACTGTCCATGA-3’ |
| Villin | 5’-ACTTCTATGGGGGCGACTG-3’ | 5’-ATGCGTCCCTTGAAGATGG-3’ |
| CYP1A1 | 5’-CTTGGACCTCTTTGGAGCTG-3’ | 5’-CGAAGGAAGAGTGTCGGAAG-3’ |
| CYP1A2 | 5’-CAGCTTCCTCATCCTCCTGCTA-3’ | 5’-AGGCTGAGCATCTCATCGCTAC-3’ |
| CYP2A6 | 5’-CCGTGTTCACCATTCACTTG-3’ | 5’-TTGCTGAATACCACGCCATA-3’ |
| CYP2C8 | 5’-GAACACCAAGCATCACTGGA-3’ | 5’-AGCAGGAGCAGGAGTCCATA-3’ |
| CYP2C9 | 5’-GGACAGAGACGACAAGCACA-3’ | 5’-CATCTGTGTAGGGCATGTGG -3’ |
| CYP2C19 | 5’-ACTTGGAGCTGGGACAGAGA-3’ | 5’-CATCTGTGTAGGGCATGTGG-3’ |
| CYP3A4 | 5’-ACATAGCCCAGCAAAGAGCAAC-3’ | 5’-GTCTGGGATGAGAGCCATCACT-3’ |
| CYP3A5 | 5’-CTTGGCTGAAGACTGCTGTG-3’ | 5’-ATAGAGGAGCACCAGGCTGA-3’ |
| ALB | 5’-CCTGCTGACTTGCCTTCATTAG-3’ | 5’-TGGCATAGCATTCATGAGGA-3’ |
| UGT1A1 | 5’-ATGCTGTGGAGTCCCAGGGC-3’ | 5’-CCATTGATCCCAAAGAGAAAACC-3’ |
| UGT1A8 | 5’-CTGCTGACCTGTGGCTTTGCT-3’ | 5’-CCATTGAGCATCGGCGAAAT-3’ |
| UGT1A10 | 5’-CCTCTTTCCTATGTCCCCAATGA-3’ | 5’-GCAACAACCAAATTGATGTGTG-3’ |
| UGT2B10 | 5’-TGACATCGTTTTTGCAGATGCTTA-3’ | 5’-CAGGTACATAGGAAGGAGGGAA-3’ |
| SULT1A3 | 5’-GGAACCCTCAGGGCTGGAG-3’ | 5’-CGTCCTTTGGGTTTCGGG-3’ |
| SULT1A1 | 5’-GTTGGCTCTGCAGGGTTTCTAGGA-3’ | 5’-CCCAAACCCCCTGCTGGCCAGCACCC-3’ |
